# Supplementary material for: Modeling glioblastoma heterogeneity as a dynamic network of cell states
Source: Mol Syst Biol. 2021 Sep 16;17(9):e10105. doi: 10.15252/msb.202010105 (PMC8444284; doi:10.15252/msb.202010105)
Supplement: Supplementary file 6 — Source Data for Figure 5 [file MSB-17-e10105-s004.zip › Figure5A_sourcedata/GSEA_3071/nosigdb_stateIV.GseaPreranked.1622795164626/RICHARDS_ZHONG_NATURE_2018_NPCS_UPREG.html]

Details for gene set RICHARDS\_ZHONG\_NATURE\_2018\_NPCS\_UPREG[GSEA]

|  || Dataset | state53071 |
| Phenotype | NoPhenotypeAvailable |
| Upregulated in class | na\_neg |
| GeneSet | RICHARDS\_ZHONG\_NATURE\_2018\_NPCS\_UPREG |
| Enrichment Score (ES) | -0.631511 |
| Normalized Enrichment Score (NES) | -3.3996599 |
| Nominal p-value | 0.0 |
| FDR q-value | 0.0 |
| FWER p-Value | 0.0 |
Table: GSEA Results Summary

  

Fig 1: Enrichment plot: RICHARDS\_ZHONG\_NATURE\_2018\_NPCS\_UPREG      
 Profile of the Running ES Score & Positions of GeneSet Members on the Rank Ordered List

  

| PROBE | GENE SYMBOL | GENE\_TITLE | RANK IN GENE LIST | RANK METRIC SCORE | RUNNING ES | CORE ENRICHMENT || 1 | ID4 |  |  | 7 | 2.594 | 0.0240 | No |
| 2 | ZFHX4 |  |  | 166 | 1.335 | 0.0065 | No |
| 3 | SOX9 |  |  | 214 | 1.223 | 0.0094 | No |
| 4 | DACH1 |  |  | 303 | 1.033 | 0.0025 | No |
| 5 | TGIF1 |  |  | 402 | 0.906 | -0.0076 | No |
| 6 | MDK |  |  | 495 | 0.819 | -0.0173 | No |
| 7 | NOTCH1 |  |  | 666 | 0.678 | -0.0436 | No |
| 8 | CDON |  |  | 764 | 0.622 | -0.0562 | No |
| 9 | CDCA7 |  |  | 818 | 0.596 | -0.0606 | No |
| 10 | PAX6 |  |  | 1275 | 0.445 | -0.1444 | No |
| 11 | CREB5 |  |  | 1546 | 0.379 | -0.1929 | No |
| 12 | SFRP1 |  |  | 1979 | 0.301 | -0.2734 | No |
| 13 | PTTG1 |  |  | 2470 | -0.266 | -0.3655 | No |
| 14 | RNASEH2A |  |  | 2595 | -0.282 | -0.3867 | No |
| 15 | UCP2 |  |  | 2602 | -0.283 | -0.3851 | No |
| 16 | DEPDC1B |  |  | 2744 | -0.300 | -0.4094 | No |
| 17 | CNTLN |  |  | 2960 | -0.327 | -0.4478 | No |
| 18 | SMC4 |  |  | 3035 | -0.337 | -0.4588 | No |
| 19 | MCM2 |  |  | 3240 | -0.366 | -0.4946 | No |
| 20 | TUBA1B |  |  | 3357 | -0.383 | -0.5133 | No |
| 21 | GNG5 |  |  | 3481 | -0.405 | -0.5331 | No |
| 22 | PAICS |  |  | 3594 | -0.427 | -0.5505 | No |
| 23 | CKAP2 |  |  | 3633 | -0.433 | -0.5536 | No |
| 24 | KNSTRN |  |  | 3646 | -0.435 | -0.5517 | No |
| 25 | KIF22 |  |  | 3832 | -0.472 | -0.5828 | No |
| 26 | MIS18BP1 |  |  | 3913 | -0.490 | -0.5935 | No |
| 27 | CENPF |  |  | 4003 | -0.511 | -0.6057 | No |
| 28 | WDR34 |  |  | 4022 | -0.518 | -0.6041 | No |
| 29 | ITGB3BP |  |  | 4071 | -0.531 | -0.6082 | No |
| 30 | CCNB2 |  |  | 4103 | -0.542 | -0.6089 | No |
| 31 | RCN1 |  |  | 4221 | -0.573 | -0.6259 | Yes |
| 32 | HMGB2 |  |  | 4243 | -0.578 | -0.6243 | Yes |
| 33 | BUB1 |  |  | 4246 | -0.578 | -0.6190 | Yes |
| 34 | BUB3 |  |  | 4249 | -0.580 | -0.6138 | Yes |
| 35 | VIM |  |  | 4255 | -0.582 | -0.6090 | Yes |
| 36 | MCM6 |  |  | 4273 | -0.590 | -0.6066 | Yes |
| 37 | SPAG5 |  |  | 4290 | -0.595 | -0.6038 | Yes |
| 38 | KIF15 |  |  | 4324 | -0.603 | -0.6043 | Yes |
| 39 | KPNA2 |  |  | 4361 | -0.621 | -0.6052 | Yes |
| 40 | C19orf48 |  |  | 4427 | -0.644 | -0.6115 | Yes |
| 41 | RPA3 |  |  | 4482 | -0.670 | -0.6153 | Yes |
| 42 | H2AFX |  |  | 4495 | -0.677 | -0.6110 | Yes |
| 43 | DHFR |  |  | 4517 | -0.688 | -0.6084 | Yes |
| 44 | DTYMK |  |  | 4566 | -0.715 | -0.6106 | Yes |
| 45 | LDHA |  |  | 4574 | -0.722 | -0.6049 | Yes |
| 46 | TUBB4B |  |  | 4575 | -0.722 | -0.5979 | Yes |
| 47 | PBK |  |  | 4593 | -0.730 | -0.5940 | Yes |
| 48 | CKS2 |  |  | 4600 | -0.733 | -0.5880 | Yes |
| 49 | BIRC5 |  |  | 4618 | -0.745 | -0.5840 | Yes |
| 50 | DUT |  |  | 4642 | -0.762 | -0.5810 | Yes |
| 51 | NUSAP1 |  |  | 4654 | -0.765 | -0.5757 | Yes |
| 52 | HSPB1 |  |  | 4701 | -0.797 | -0.5768 | Yes |
| 53 | MCM7 |  |  | 4704 | -0.800 | -0.5693 | Yes |
| 54 | GTSE1 |  |  | 4722 | -0.808 | -0.5647 | Yes |
| 55 | MELK |  |  | 4758 | -0.835 | -0.5633 | Yes |
| 56 | KIF11 |  |  | 4760 | -0.835 | -0.5553 | Yes |
| 57 | C21orf58 |  |  | 4767 | -0.838 | -0.5483 | Yes |
| 58 | CKAP2L |  |  | 4769 | -0.839 | -0.5403 | Yes |
| 59 | NUF2 |  |  | 4782 | -0.847 | -0.5343 | Yes |
| 60 | ASPM |  |  | 4789 | -0.851 | -0.5272 | Yes |
| 61 | TMPO |  |  | 4790 | -0.851 | -0.5188 | Yes |
| 62 | MCM5 |  |  | 4792 | -0.853 | -0.5107 | Yes |
| 63 | CENPN |  |  | 4796 | -0.858 | -0.5029 | Yes |
| 64 | HELLS |  |  | 4819 | -0.882 | -0.4985 | Yes |
| 65 | KIF23 |  |  | 4825 | -0.891 | -0.4908 | Yes |
| 66 | MCM3 |  |  | 4834 | -0.901 | -0.4835 | Yes |
| 67 | MKI67 |  |  | 4836 | -0.902 | -0.4749 | Yes |
| 68 | PRC1 |  |  | 4846 | -0.912 | -0.4677 | Yes |
| 69 | SMC2 |  |  | 4847 | -0.912 | -0.4588 | Yes |
| 70 | DLGAP5 |  |  | 4857 | -0.922 | -0.4515 | Yes |
| 71 | UHRF1 |  |  | 4859 | -0.924 | -0.4427 | Yes |
| 72 | ORC6 |  |  | 4865 | -0.931 | -0.4345 | Yes |
| 73 | PRR11 |  |  | 4868 | -0.934 | -0.4258 | Yes |
| 74 | GMNN |  |  | 4873 | -0.939 | -0.4174 | Yes |
| 75 | CDCA3 |  |  | 4874 | -0.940 | -0.4082 | Yes |
| 76 | TMEM98 |  |  | 4880 | -0.945 | -0.3999 | Yes |
| 77 | CENPM |  |  | 4881 | -0.947 | -0.3906 | Yes |
| 78 | GINS2 |  |  | 4897 | -0.962 | -0.3841 | Yes |
| 79 | TACC3 |  |  | 4900 | -0.966 | -0.3751 | Yes |
| 80 | CDCA4 |  |  | 4902 | -0.967 | -0.3658 | Yes |
| 81 | FBXO5 |  |  | 4910 | -0.978 | -0.3576 | Yes |
| 82 | TAGLN2 |  |  | 4917 | -0.983 | -0.3491 | Yes |
| 83 | H2AFZ |  |  | 4919 | -0.983 | -0.3397 | Yes |
| 84 | SNRPB |  |  | 4931 | -0.996 | -0.3321 | Yes |
| 85 | MCM4 |  |  | 4947 | -1.011 | -0.3251 | Yes |
| 86 | CCNB1 |  |  | 4962 | -1.032 | -0.3177 | Yes |
| 87 | CLIC1 |  |  | 4964 | -1.035 | -0.3078 | Yes |
| 88 | CENPW |  |  | 4978 | -1.056 | -0.3000 | Yes |
| 89 | ATAD2 |  |  | 4980 | -1.058 | -0.2898 | Yes |
| 90 | RRM1 |  |  | 4990 | -1.074 | -0.2811 | Yes |
| 91 | UBE2T |  |  | 5000 | -1.091 | -0.2722 | Yes |
| 92 | MAD2L1 |  |  | 5007 | -1.105 | -0.2625 | Yes |
| 93 | SPC25 |  |  | 5012 | -1.112 | -0.2524 | Yes |
| 94 | FEN1 |  |  | 5014 | -1.113 | -0.2417 | Yes |
| 95 | AURKB |  |  | 5016 | -1.118 | -0.2310 | Yes |
| 96 | CDCA5 |  |  | 5023 | -1.133 | -0.2211 | Yes |
| 97 | KIF2C |  |  | 5028 | -1.150 | -0.2106 | Yes |
| 98 | TOP2A |  |  | 5029 | -1.152 | -0.1993 | Yes |
| 99 | IGF2BP1 |  |  | 5036 | -1.166 | -0.1891 | Yes |
| 100 | TPX2 |  |  | 5049 | -1.184 | -0.1798 | Yes |
| 101 | CCNA2 |  |  | 5051 | -1.186 | -0.1684 | Yes |
| 102 | NDC80 |  |  | 5055 | -1.200 | -0.1573 | Yes |
| 103 | PCNA |  |  | 5060 | -1.214 | -0.1462 | Yes |
| 104 | CDC20 |  |  | 5082 | -1.249 | -0.1380 | Yes |
| 105 | MFAP2 |  |  | 5087 | -1.259 | -0.1265 | Yes |
| 106 | CENPK |  |  | 5091 | -1.267 | -0.1147 | Yes |
| 107 | UBE2C |  |  | 5106 | -1.314 | -0.1045 | Yes |
| 108 | ZWINT |  |  | 5115 | -1.321 | -0.0931 | Yes |
| 109 | CKS1B |  |  | 5121 | -1.338 | -0.0810 | Yes |
| 110 | HJURP |  |  | 5125 | -1.344 | -0.0685 | Yes |
| 111 | CENPU |  |  | 5159 | -1.486 | -0.0603 | Yes |
| 112 | CDK1 |  |  | 5164 | -1.512 | -0.0463 | Yes |
| 113 | PHGDH |  |  | 5170 | -1.528 | -0.0323 | Yes |
| 114 | CLSPN |  |  | 5202 | -1.663 | -0.0221 | Yes |
| 115 | RRM2 |  |  | 5232 | -1.875 | -0.0093 | Yes |
| 116 | HIST1H4C |  |  | 5249 | -2.081 | 0.0079 | Yes |
Table: GSEA details [plain text format]

  

Fig 2: RICHARDS\_ZHONG\_NATURE\_2018\_NPCS\_UPREG: Random ES distribution      
 Gene set null distribution of ES for **RICHARDS\_ZHONG\_NATURE\_2018\_NPCS\_UPREG**
